# Single-Gene Congenic Strain Reveals the Effect of *Zbtb16* on Dexamethasone-Induced Insulin Resistance

**DOI:** 10.3389/fendo.2018.00185

**Published:** 2018-04-20

**Authors:** Michaela Krupková, František Liška, Ludmila Kazdová, Lucie Šedová, Adéla Kábelová, Drahomíra Křenová, Vladimír Křen, Ondřej Šeda

**Affiliations:** ^1^The First Faculty of Medicine, Institute of Biology and Medical Genetics, Charles University, The General Teaching Hospital, Prague, Czechia; ^2^Centre for Experimental Medicine, Institute for Clinical and Experimental Medicine, Prague, Czechia; ^3^Laboratory of Transgenic Models of Diseases, Division BIOCEV, Institute of Molecular Genetics of the Czech Academy of Sciences, v.v.i., Vestec, Prague, Czechia

**Keywords:** ZBTB16, dexamethasone, rat models, congenic strain, pharmacogenetics and pharmacogenomics, insulin resistance

## Abstract

**Background:**

Glucocorticoids (GCs) are potent therapeutic agents frequently used for treatment of number of conditions, including hematologic, inflammatory, and allergic diseases. Both their therapeutic and adverse effects display significant interindividual variation, partially attributable to genetic factors. We have previously isolated a seven-gene region of rat chromosome 8 sensitizing to dexamethasone (DEX)-induced dyslipidemia and insulin resistance (IR) of skeletal muscle. Using two newly derived congenic strains, we aimed to investigate the effect of one of the prime candidates for this pharmacogenetic interaction, the *Zbtb16* gene.

**Methods:**

Adult male rats of SHR-*Lx*.PD5^PD-^*^Zbtb16^* (*n* = 9) and SHR-*Lx*.PD5^SHR-^*^Zbtb16^* (*n* = 8) were fed standard diet (STD) and subsequently treated with DEX in drinking water (2.6 µg/ml) for 3 days. The morphometric and metabolic profiles of both strains including oral glucose tolerance test, triacylglycerols (TGs), free fatty acids, insulin, and C-reactive protein levels were assessed before and after the DEX treatment. Insulin sensitivity of skeletal muscle and visceral adipose tissue was determined by incorporation of radioactively labeled glucose.

**Results:**

The differential segment of SHR-*Lx*.PD5^SHR-^*^Zbtb16^* rat strain spans 563 kb and contains six genes: *Htr3a, Htr3b, Usp28, Zw10, Tmprss5*, and part of *Drd2*. The SHR-*Lx*.PD5^PD-^*^Zbtb16^* minimal congenic strain contains only *Zbtb16* gene on SHR genomic background and its differential segment spans 254 kb. Total body weight was significantly increased in SHR-*Lx*.PD5^PD-^*^Zbtb16^* strain compared with SHR-Lx.PD5^SHR-^*^Zbtb16^*, however, no differences in the weights of adipose tissue depots were observed. While STD-fed rats of both strains did not show major differences in their metabolic profiles, after DEX treatment the SHR-*Lx*.PD5^PD-^*^Zbtb16^* congenic strain showed increased levels of TGs, glucose, and blunted inhibition of lipolysis by insulin. Both basal and insulin-stimulated incorporation of radioactively labeled glucose into skeletal muscle glycogen were significantly reduced in SHR-*Lx*.PD5^PD-^*^Zbtb16^* strain, but the insulin sensitivity of adipose tissue was comparable between the two strains.

**Conclusion:**

The metabolic disturbances including impaired glucose tolerance, dyslipidemia, and IR of skeletal muscle observed after DEX treatment in the congenic SHR-*Lx*.PD5^PD-^*^Zbtb16^* reveal the *Zbtb1*6 locus as a possible sensitizing factor for side effects of GC therapy.

## Introduction

Glucocorticoids (GCs) are potent therapeutic agents frequently used for treatment of number of conditions, including hematologic, inflammatory, and allergic diseases (1). The therapeutic effect of GCs administration has been repeatedly shown to vary interindividually due to numerous factors like gender, age, and particularly, genetic constitution of the treated individual. One extreme of the range of varied responses is a near- to complete failure to respond to GC therapy, described as GC insensitivity or resistance (2, 3). Similar variety is observed in unwanted, side effects accompanying GCs therapy—lower bone mineral density and osteoporosis, diabetes, dyslipidemia, obesity, impaired wound healing, or muscle wasting (4). Manifestation of the adverse effects is, to certain extent, dependent on the therapeutic dose and time of treatment; however, pharmacogenetic interactions have been implicated as substantial contributing factors (5, 6). Variation in genes coding for proteins directly involved in GC genomic actions were shown to alter GC sensitivity, namely, the GC receptor (*NR3C1*), recently summarized by Nicolaides and Charmandari (7). Clearly, other genes can also mediate the clinically relevant adverse effects or changes in response to GC. For instance, genome-wide association study (GWAS) of response to inhaled GCs in asthma patients identified a polymorphism in glucocorticoid-induced transcript 1 gene (*GLCCI1*) and further replicated the lower response of individuals carrying two copies of the variant allele in four independent cohorts (8). In another GWAS performed in acute lymphoblastic leukemia patients, dexamethasone (DEX)-induced pleiotropic side effects were significantly associated with variation in *F2RL1* (F2R like trypsin receptor 1) gene (9). In an experimental, genetically designed model strain set, we showed previously that several genomic loci affect sensitivity toward DEX-induced unfavorable changes in carbohydrate and lipid metabolism (10). Later, we reported that a presence of defective *Cd36/Fat*-containing genomic segment substantially blunted DEX-induced glucose intolerance and dyslipidemia (11). Also, we have isolated a small region of rat chromosome 8, which sensitized its carrier toward DEX-induced, muscle-specific insulin resistance (IR). In the process of positional cloning of gene(s) connected to several aspects of metabolic syndrome based on results of linkage and association studies in segregating populations and recombinant inbred strains, we derived congenic strain SHR-*Lx*.PD5 (12). This strain harbors a limited rat chromosome 8 segment of polydactylous rat (PD/Cub) origin on the genomic background of the spontaneously hypertensive rat (SHR). While SHR represents one of the most studied models of essential hypertension (13), it shows also IR, dyslipidemia, and other features of metabolic syndrome (14). By contrast, the PD/Cub strain is a model of metabolic syndrome without elevated blood pressure (15), particularly sensitive to nutritional and pharmacologic challenges (10, 16–18). We have hypothesized that a PD/Cub-derived, GC-responsive gene present within the isolated region, *Zbtb16* (zinc finger and BTB domain containing 16), is a prime candidate for the observed pharmacogenetic interaction (12). Our previous sequence analysis revealed a conservative amino acid substitution (T208S) and a 2,964-bp deletion in intron 2 of PD/Cub’s *Zbtb16* gene (19). Zbtb16 is a pleiotropic transcription factor involved, among others, in processes underlying pathogenesis of practically all major constituents of metabolic syndrome, as reviewed recently (20). In this study, we created two new models, one of them carrying only the *Zbtb16* gene from the original segment to retest its effect on DEX-induced metabolic syndrome features by comparing their metabolic profiles including global and tissue-specific insulin sensitivity.

## Materials and Methods

### Ethics Statement

All experiments were performed in agreement with the Animal Protection Law of the Czech Republic (311/1997) which is in compliance with the European Community Council recommendations for the use of laboratory animals 86/609/ECC and were evaluated and approved by the Ethical committee of the First Faculty of Medicine and the Ministry of Education, Youth and Sports of the Czech Republic (protocol ID MSMT-1461/2015-17). The health of the rats was examined daily and monitored every 1 h during the experimental procedures. There were no unexpected deaths throughout the experiment. All efforts were made to minimize suffering of the experimental animals.

### Derivation of the SHR-*Lx*.PD5^PD-*Zbtb16*^ and SHR-*Lx*.PD5^SHR-*Zbtb16*^ Congenic Strains

The SHR/OlaIpcv [SHR hereafter, RGD (21) ID no. 631848] and SHR-*Lx*.PD5 (RGD ID no. 1641851) strains were maintained at the Institute of Medical Biology and Genetics, Charles University in Prague. To derive SHR-*Lx*.PD5^PD-^*^Zbtb16^* and SHR-*Lx*.PD5^SHR-^*^Zbtb16^* congenic strains, a marker-assisted backcross breeding approach was used, as described previously (12, 22, 23). In short, SHR rats were crossed with SHR-*Lx*.PD5 rats, and the subsequent F1 hybrids were repeatedly backcrossed to SHR. The differential segments in the respective strains were fixed by intercrossing heterozygotes and selecting the progeny with homozygous SHR-*Lx*.PD5-derived chromosome 8 segments. The congenic status of the new strains was validated with a whole-genome marker scan.

### DNA Extraction and Genotyping

Rat DNA was isolated from tail samples by the modified phenol extraction method. Primer nucleotide sequences were obtained from public databases, particularly the Rat Genome Database (RGD, http://rgd.mcw.edu), or designed using Primer3 (24). Polymerase chain reaction (PCR) was used for genotyping markers polymorphic between progenitor strains. PCR was performed in 20 µl reaction volume containing 50–100 ng of genomic DNA, 1 U Taq polymerase (Fermentas), 1× KCl reaction buffer (Fermentas), 1 M betaine (Sigma-Aldrich), 1.5 mM MgCl_2_, 50 µM of each dNTP, 200 nM of each forward and reverse primers, and 5 mM cresol red (Sigma-Aldrich, for instant gel loading purpose). Cycling conditions were as follows: initial denaturation for 2 min 30 s at 93°C, then 35 cycles of denaturation at 93°C/30 s, annealing at 53°C/40 s and elongation at 72°C/50 s; 72°C/5 min final elongation. Zbtb16 2,964 bp deletion was genotyped using Phusion DNA polymerase (New England Biolabs) according to the manufacturer’s protocol, cycling conditions were as follows: initial denaturation for 30 s at 98°C, then 30 cycles of denaturation at 98°C/10 s, annealing at 63°C/30 s and elongation at 72°C/1 min 30 s; 72°C/5 min final elongation. We tested DNA from the congenic strains (SHR-*Lx*.PD5^PD-^*^Zbtb16^* and SHR-*Lx*.PD5^SHR-^*^Zbtb16^, n* = 10 per strain) and the progenitor strains SHR, SHR-*Lx*.PD5, as well as the donor of differential segment in SHR-*Lx*.PD5, the PD/Cub strain. For genotyping the microsatellites, the PCR products were separated on polyacrylamide (7–10%) gels and detected in UV light after ethidium bromide staining using Syngene G:Box (Synoptics Ltd., Cambridge, UK). For genotyping single nucleotide variants, PCR products were sequenced directly using BigDye v1.1 Cycle Sequencing kit and sequencing reactions electrophoresed using ABI PRISM 310 (Applied Biosystems). The complete set of markers used to determine the extents of differential segments is listed in Table S1 in Supplementary Material, and representative gel images are shown in Figure S1 in Supplementary Material.

### Experimental Protocol

The selection of experimental protocol and specific ages of rats was made to match our previous study (12) to replicate our findings in the newly derived congenic strains. Adult rat males were held under temperature and humidity controlled conditions on 12 h/12 h light–dark cycle. At all times, the animals had free access to food (except the single overnight fast) and water. Male SHR-*Lx*.PD5^PD-^*^Zbtb16^* (*n* = 9) and SHR-*Lx*.PD5^SHR-^*^Zbtb16^* (*n* = 8) rats were fed standard laboratory chow *ad libitum*. At the age of 5 months, the oral glucose tolerance test (OGTT) was performed after overnight fasting. Blood for determination of glycemia (Ascensia Elite Blood Glucose Meter; Bayer HealthCare, Mishawaka, IN, USA, validated by Institute of Clinical Biochemistry and Laboratory Diagnostics of the First Faculty of Medicine), insulin (ELISA kit for rat insulin assay, Mercodia, Uppsala, Sweden), free fatty acids (FFAs) (acyl-CoA oxidase-based colorimetric kit, Roche Diagnostics GmbH, Mannheim, Germany), and triacylglycerols (TGs) (standard enzymatic method, Erba-Lachema, Brno, Czech Republic), was drawn from the tail at intervals of 0, 30, 60, 120, and 180 min after the intragastric glucose administration to conscious rats (3 g/kg body weight, 30% aqueous solution). Seven days after the original OGTT, all rats were administered DEX (Dexamed, Medochemie) in drinking water (2.6 µg/ml) for 3 days as described previously (11, 12), and blood was drawn in fed state for determination of TGs, FFAs, insulin, and C-reactive protein (ELISA kit, Alpha Diagnostics International, San Antonio, TX, USA). After an overnight fast, the OGTT was repeated including all the measurements described earlier. Then, the animals were sacrificed, and the total weight and weight of heart, liver, kidneys, adrenals, epididymal, and retroperitoneal fat pads were determined.

For determination of TG content in the liver, tissue was powdered under liquid N_2_ and extracted over 16 h in chloroform:methanol after which 2% KH_2_PO_4_ was added and the solution centrifuged. The organic phase was removed and evaporated under N_2_. The resulting pellet was dissolved in isopropyl alcohol, and the TG content was determined by enzymatic assay (Erba-Lachema, Brno, Czech Republic).

### Basal- and Insulin-Stimulated Glycogen Synthesis in Muscle, and Glucose Utilization in Isolated White Adipose Tissue

Diaphragmatic muscles were immediately after excision incubated in Krebs-Ringer bicarbonate buffer (pH 7.4) containing 0.1 μCi of [U-^14^C] glucose, 5 mmol/l of unlabeled glucose, and 2.5 mg/ml of bovine serum albumin (Fraction V; Sigma-Aldrich, St. Louis, MO, USA) in the presence (250 μU/ml) or absence of insulin in incubation media. All incubations were performed in a 95% O_2_ + 5% CO_2_ atmosphere in sealed vials at 37°C in a shaking water bath. After 2 h incubation tissue glycogen was extracted, and basal and insulin-stimulated incorporation of [U-^14^C] glucose was determined.

Pieces of epididymal fat were rapidly dissected and incubated for 2 h in Krebs-Ringer bicarbonate buffer with 5 mmol/l glucose, 0.1 μCi [U-^14^C] glucose/ml (UVVR, Prague, Czech Republic), and 2% bovine serum albumin, gaseous phase 95% O_2_ + 5% CO_2_ in the presence (250 μU/ml) or absence of insulin in incubation media. All incubations were performed at 37°C in sealed vials in a shaking water bath.

We estimated incorporation of [U-^14^C] glucose into neutral lipids. Briefly, fat was removed from incubation medium, rinsed in saline, and immediately placed into chloroform. Pieces of tissue were dissolved using a teflon pestle homogenizer, methanol was added (chloroform:methanol 2:1), and lipids were extracted at 4°C overnight. The remaining tissue was removed, KH_2_PO_4_ was added, and the clear extract was taken for further analysis. An aliquot was evaporated, reconstituted in scintillation liquid, and radioactivity measured by scintillation counting. Incremental glucose utilization was calculated as the difference between the insulin-stimulated and basal incorporation of glucose into neutral lipids.

### Statistical Analysis

All statistical analyses were performed using STATISTICA 13.2. Unpaired Student’s *t*-test was used for comparison of morphometric traits and metabolic of the SHR-*Lx*.PD5^PD-^*^Zbtb16^* vs. SHR-*Lx*.PD5^SHR-^*^Zbtb16^* strains. Repeated measures ANOVA was used for comparison of repeatedly measured parameters. The differences were considered statistically significant when *P* < 0.05.

## Results

### Genomic Characterization of the SHR-*Lx*.PD5 Congenic Substrains

**Figure 1:**
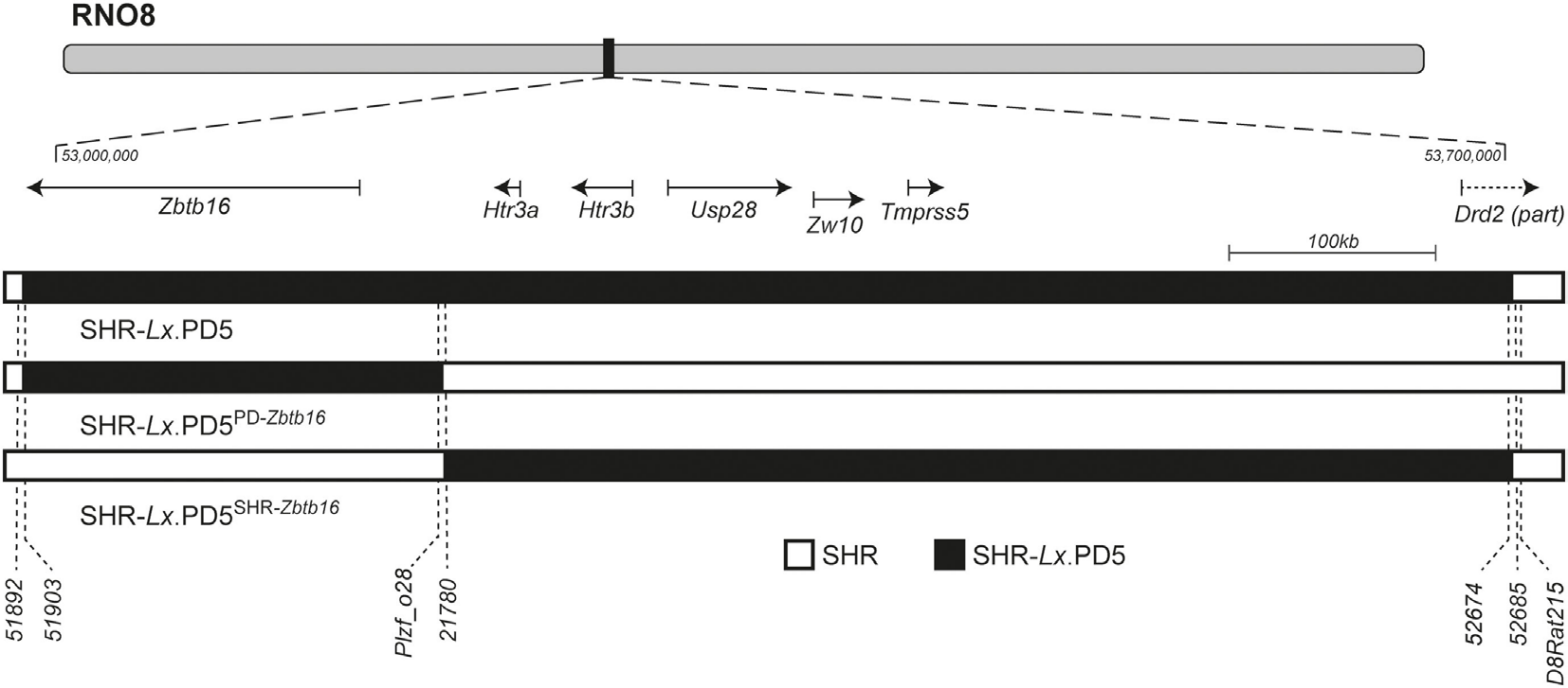
The rat chromosome 8 (RNO8) differential segments in SHR-*Lx*.PD5 congenic strains. The schematic representation of RNO8 with indication of the relative position and extent of the differential segments in the congenic strains is shown at the top. The portrayed region corresponds to the interval between 53 and 53.7 Mb [based on rat genome assembly: Rnor_6.0 (GCA_000001895.4)]. The gene track shows the RefSeq genes present within the differential segments: *Zbtb16* (zinc finger and BTB domain containing 16), *Htr3a* (5-hydroxytryptamine receptor 3A), *Htr3b* (5-hydroxytryptamine receptor 3B), *Usp28* (ubiquitin specific peptidase 28), *Zw10* (zw10 kinetochore protein), *Tmprss5* (transmembrane protease, serine 5), and *Drd2* (dopamine receptor D2). SHR-*Lx*.PD5^PD-^*^Zbtb16^* harbors only 254 kb segment of the PD/Cub origin (black bar) including only the *Zbtb16* gene on SHR genomic background (open bar) while the SHR-*Lx*.PD5^SHR-^*^Zbtb16^* carries the remaining 563 kb-long part of the original SHR-*Lx*.PD5 segment (black bar), while the rest of RNO8 and its genome is of the SHR origin (open bar). The position of selected polymorphic markers is shown under the chromosome schemes. The complete annotation of markers used for genotypic characterization of the differential segments is given in Table S1 in Supplementary Material.

The derivation of the original SHR-*Lx*.PD5, initial genomic characterization and detailed genetic scan including *in silico* analysis and manual sequencing of the coding parts of the genes and selected conserved non-coding regions was reported previously (12, 19). We utilized the same extensive set of polymorphic markers to characterize the span of rat chromosome 8 (RNO8) differential segments in the newly derived SHR-*Lx*.PD5^SHR-^*^Zbtb16^* and SHR-*Lx*.PD5^PD-^*^Zbtb16^* congenic strains. As shown in Figure 1, the SHR-*Lx*.PD5^PD-^*^Zbtb16^* harbors a 254 kb segment of the PD/Cub origin including only the *Zbtb16* gene on SHR genomic background while the SHR-*Lx*.PD5^SHR-^*^Zbtb16^* carries the SHR variant of the *Zbtb16* gene together with remaining 563 kb-long part of the original SHR-*Lx*.PD5 segment. Since we did not identify any additional non-SHR alleles throughout the genomic scan of polymorphic markers in either of the new strains, the congenic status of SHR-*Lx*.PD5^SHR-^*^Zbtb16^* and SHR-*Lx*.PD5^PD-^*^Zbtb16^* can be considered as validated.

### Morphometric and Metabolic Profile Comparison

Both before an after DEX administration, the SHR-*Lx*.PD5^PD-^*^Zbtb16^* rats were significantly heavier in comparison with SHR-*Lx*.PD5^SHR-^*^Zbtb16^* strain. Weights of liver, heart, kidneys, and the visceral and retroperitoneal adipose tissue depots were higher in SHR-*Lx*.PD5^PD-^*^Zbtb16^* rats (Table 1). When corrected for body weight, morphometric parameters did not differ between the two strains except for liver, which remained significantly heavier in SHR-*Lx*.PD5^PD-^*^Zbtb16^*. In fed state, we observed significantly higher TG concentrations in SHR-*Lx*.PD5^PD-^*^Zbtb16^* in combination with lower TG content in liver when compared with SHR-*Lx*.PD5^SHR-^*^Zbtb16^*. The concentrations of FFAs, insulin, TG in muscle, and serum CRP were comparable in the two strains (Table 1).

**Table 1:** Morphometric and metabolic profile of the congenic rat strains.

Trait	SHR-*Lx*.PD5^PD-^*^Zbtb16^*	SHR-*Lx*.PD5^SHR-^*^Zbtb16^*	*P*
*N*	9	8	
Body weight (pre-DEX), g	299 ± 8	276 ± 7	**0.040**
Body weight, g	287 ± 7	265 ± 4	**0.030**
Liver weight, g	11.9 ± 0.7	9.0 ± 0.5	**0.004**
Heart weight, g	1.19 ± 0.03	1.07 ± 0.02	**0.003**
Kidney weight, g	2.52 ± 0.10	2.18 ± 0.09	**0.018**
Adrenals weight, mg	55.0 ± 5.5	54.0 ± 6.8	*0.91*
EFP weight, g	2.27 ± 0.10	1.71 ± 0.16	**0.010**
RFP weight, g	1.10 ± 0.03	0.82 ± 0.07	**0.046**
TG (fed), mmol/l	2.08 ± 0.13	1.64 ± 0.10	**0.015**
FFA (fed), mmol/l	0.61 ± 0.03	0.59 ± 0.06	*0.77*
Insulin (fed), nmol/l	0.32 ± 0.04	0.32 ± 0.10	*0.99*
TG in liver, μmol/g	4.95 ± 0.55	6.48 ± 0.37	**0.026**
TG in muscle, μmol/g	0.85 ± 0.29	0.90 ± 0.20	*0.94*
CRP, mg/l	526 ± 54	449 ± 52	*0.52*

*Morphometric and metabolic profile of SHR-Lx.PD5^PD-^^Zbtb16^ vs. SHR-Lx.PD5^SHR-^^Zbtb16^ male rats after 3-day administration of dexamethasone*.

*Values are shown as mean ± SEM*.

*The significance levels of pair-wise, inter-strain comparisons between the two strains are shown in third column*.

*Significant differences highlighted in bold, non-significant ones in italics*.

*EFP, epididymal fat pad; RFP, retroperitoneal fat pad; FFA, free fatty acid; TG, triacylglycerol; CRP, C-reactive protein; DEX, dexamethasone*.

**Figure 2:**
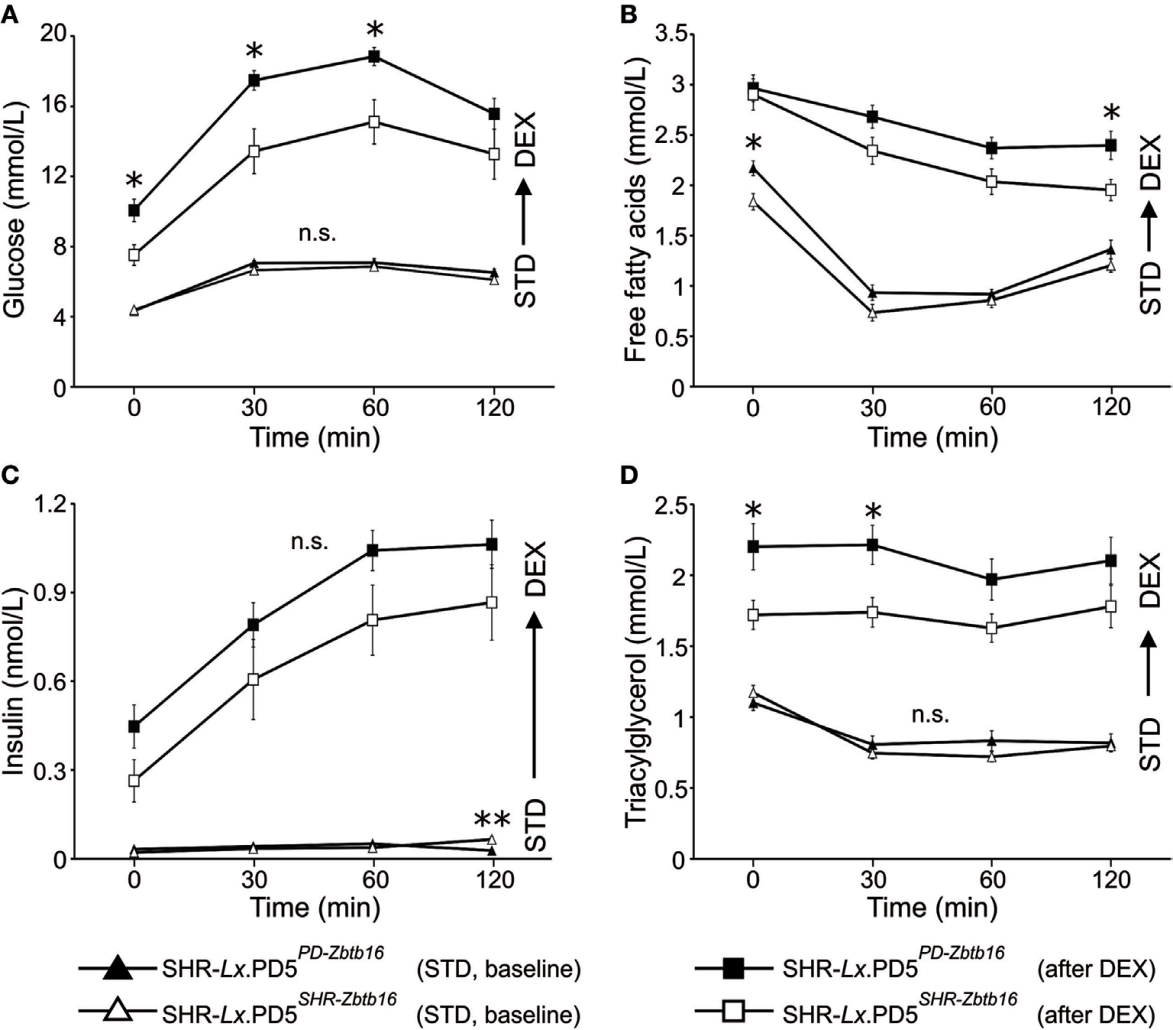
The oral glucose tolerance test (OGTT). Time course of glucose **(A)**, free fatty acids **(B)**, insulin **(C)**, and triacylglycerols **(D)** during OGTT is shown for standard diet-fed SHR-*Lx*.PD5^PD-^*^Zbtb16^* (black triangles, *n* = 9) and SHR-*Lx*.PD5^SHR-^*^Zbtb16^* (open triangles, *n* = 8) male rats. The procedure was then repeated in the same animals after administration of dexamethasone (values for SHR-*Lx*.PD5^PD-^*^Zbtb16^* shown as black squares, SHR-*Lx*.PD5^SHR-^*^Zbtb16^* shown in open squares). Adjusted statistical significance levels using the repeated measured ANOVA are indicated for differences between the strains under identical conditions as *P < 0.05.

When fed standard diet (STD), the glycemia time courses during the OGTT were nearly identical (Figure 2A). The levels of insulin also did not differ until the second hour of the test, when the SHR-*Lx*.PD5^PD-^*^Zbtb16^* exhibited lower values compared with SHR-*Lx*.PD5^SHR-^*^Zbtb16^* (Figure 2C). The fasting concentrations of FFA were significantly higher in SHR-*Lx*.PD5^PD-^*^Zbtb16^*, yet in the remaining time points of OGTT the two strains did not diverge. No significant differences were observed for TG concentrations. After the DEX challenge (performed in the identical animals assessed under STD conditions), fasting glycemia and glucose concentrations during the first hour of OGTT were significantly higher in SHR-*Lx*.PD5^PD-^*^Zbtb16^* in comparison with SHR-*Lx*.PD5^SHR-^*^Zbtb16^* with no difference in insulin levels all the way through the test. The suppression of FFA levels by insulin was less effective in SHR-*Lx*.PD5^PD-^*^Zbtb16^* as evidenced in Figure 2B, ending in significantly higher FFA concentrations 120 min after the glucose bolus administration. Contrasting with baseline conditions, SHR-*Lx*.PD5^PD-^*^Zbtb16^* exhibited elevated TG at fasting state and 30 min into the OGTT, then the values converged (Figure 2D).

### Insulin Sensitivity of Visceral Adipose Tissue and Skeletal Muscle

**Figure 3:**
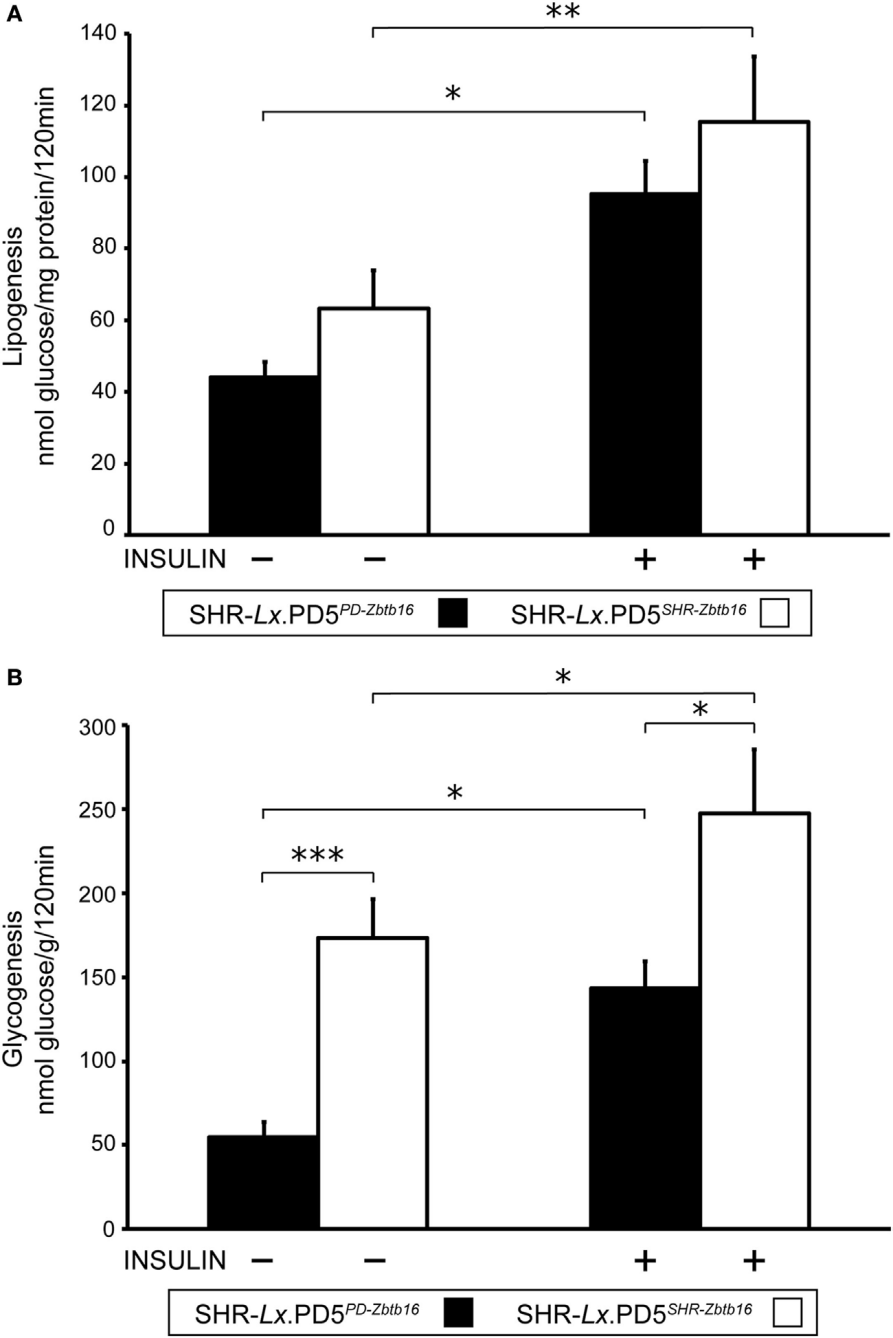
Insulin sensitivity of peripheral tissues in the SHR-*Lx*.PD5^PD-^*^Zbtb16^* and SHR-*Lx*.PD5^SHR-^*^Zbtb16^* male rats. Basal- and insulin-stimulated [U-^14^C] glucose incorporation into total lipids of epididymal (visceral) adipose tissue [lipogenesis **(A)**] and into glycogen of the diaphragm [glycogenesis **(B)**] in the SHR-*Lx*.PD5^PD-^*^Zbtb16^* (full bars) and SHR-*Lx*.PD5^SHR-^*^Zbtb16^* (open bars) are shown. Data are expressed as mean ± SEM (*n* = 8 individual rats/group). Adjusted statistical significance levels using the unpaired *t*-test are indicated as *P < 0.05; **P < 0.01; and ***P < 0.001.

While the insulin sensitivity of visceral adipose tissue did not differ between the two congenic strains, both basal and insulin-stimulated incorporation of radioactively labeled glucose into skeletal muscle glycogen were substantially reduced in SHR-*Lx*.PD5^PD-^*^Zbtb16^* in comparison with SHR-*Lx*.PD5^SHR-^*^Zbtb16^* (Figure 3).

## Discussion

In a follow-up to previous identification of limited genomic region responsible for DEX-induced dyslipidemia and muscle-specific IR, we present in this study further evidence that GC-inducible transcription factor Zbtb16 (25) plays a major role in such sensitization. Zbtb16, also known as Plzf (promyelocytic leukemia zinc finger), belongs to POK (POZ and Krüppel zinc finger) family of proteins and is involved in plethora of physiological processes including maintenance of spermatogenesis (26), stem cell self-renewal (27), hematopoiesis (28), limb development (29, 30) and regulation of metabolism (31, 32). The gene is quite conserved in mammals, the human and rat ZBTB16/Zbtb16 proteins show 96% identity over 673 amino acids constituting the protein. The new, single-gene congenic strain carrying the variant of *Zbtb16* gene (3 kb intronic deletion + T208S substitution) showed increased plasma TG and diminished insulin sensitivity of skeletal muscle tissue. Both these observations correspond to the original findings in parental strain SHR-*Lx*.PD5 (with seven genes in its differential segment, including *Zbtb16*) when compared with SHR (12). Reduced visceral adiposity observed in the previous study was not confirmed despite the matching difference trend (*P* = 0.06). The concept of DEX-induced IR of muscle tissue is well known, and several mechanisms have been proposed to convey this effect including direct effects on glucose transporter GLUT4 trafficking (33), increase in glycogen synthase phosphorylation together with decrease in expression and insulin-stimulated phosphorylation of protein kinase B (34) or dysregulation of intramyocellular lipid metabolism (35). To the best of our knowledge, involvement of *Zbtb16* in this process has not been reported so far. We have recently reviewed in detail the pleiotropic connections of *Zbtb16* to the facets of metabolic syndrome, including IR and dyslipidemia (20). Zbtb16 is strongly induced by GCs (36) and at the same time it negatively regulates the insulin signaling pathway by decreasing the phosphorylation of IRS1, Akt, and FoxO1 in normal mice (32). This presents potential basis for a cross-talk involving DEX, Zbtb16, and insulin sensitivity of muscle, somewhat similar to the one reported for the role of Zbtb16 in regulation of hepatic gluconeogenesis previously. In the suggested model, Zbtb16 acts as a downstream effector of the PGC-1α/GR complex, activated by gluconeogenic signals. Activated PGC-1α/GR then induces Zbtb16 expression, triggering hepatic gluconeogenesis and, at the same time, negatively regulating the insulin signaling pathway (32). The insulin sensitivity of skeletal muscle determined repeatedly in DEX-treated SHR [unpublished data and Ref. (12)] is comparable to that of SHR-*Lx*.PD5^SHR-^*^Zbtb16^* strain (SHR basal 121 ± 13; insulin-stimulated 226 ± 25 nmol glucose/g/120 min vs. SHR-*Lx*.PD5^SHR-^*^Zbtb16^* basal 173 ± 23; insulin-stimulated 247 ± 38 nmol glucose/g/120 min) contrasting with decreased sensitivity in the single-gene congenic strain with the PD/Cub mutant allele of *Zbtb16* gene as well as the original SHR-*Lx*.PD5 strain (SHR-*Lx*.PD5 basal 82 ± 6; insulin-stimulated 142 ± 10 nmol glucose/g/120 min vs. SHR-*Lx*.PD5^PD-^*^Zbtb16^* basal 55 ± 9; insulin-stimulated 144 ± 16 nmol glucose/g/120 min). In skeletal muscle, GCs were shown to cause IR by reducing transcription of IRS1, while increasing expression of proteins that interfere with insulin action, including protein tyrosine phosphatase type 1B, leukocyte common antigen-related protein (LAR) and p38MAPK (37). However, over 170 potential GC targets have been identified in muscle (38) affecting not only insulin sensitivity but also protein metabolism with effects extending to other organs, as documented in the muscle-specific GR knockout animals (39). While interactions between Zbtb16 and several of the implicated genes have been described, the identification of causal perturbed pathways in the SHR-*Lx*.PD5^PD-^*^Zbtb16^* remains to be established. The enhanced TG storage in liver due to GC administration (40) was more prominent in the SHR-*Lx*.PD5^SHR-^*^Zbtb16^* strain. In an experimental model with TALEN (transcription activator-like effector nuclease)-mediated *Zbtb16* knockout, the heterozygous SHR-*Plzf*^+/−^ animals showed decreased concentrations of TG and cholesterol in liver and plasma (41). Hepatic *Zbtb16* is upregulated in several murine models showing severe hepatic steatosis (32). However, given the higher fasting and satient plasma concentrations of TG in SHR-*Lx*.PD5^PD-^*^Zbtb16^*, we can speculate that DEX-induced activation of liver *Zbtb16* in the single congenic strain could cause a decrease of phosphorylation of FoxO1 (32), which in turn activated both gluconeogenesis (leading to increased glycemia) and increased expression of microsomal triglyceride transfer protein, augmenting very low-density lipoprotein, TG-rich particles assembly and secretion (42). The reduced total body weight and relative weight of liver in the original SHR-*Lx*.PD5 strain compared with SHR is consistent with the morphometric profile of the strain without the variant *Zbtb16* allele, i.e., the SHR-*Lx*.PD5^SHR-^*^Zbtb16^* strain. Although at this point it is impossible to pinpoint any of the six genes present in the differential segment as a major contributor, the serotonin receptor *Htr3a* was recently shown to modulate energy expenditure and weight gain *via* adipose tissue (43) and can be thus considered as a potential candidate. The limitations of this study include the use of only male rats as sex-specific genetic architecture of the metabolic syndrome and its components was demonstrated (44). Also, as the experimental protocol was set up to mimic the original set of experiments comparing SHR rats to SHR-*Lx*.PD5 congenic, only one dose of DEX and a short-term administration in 5-month-old animals was used. It is possible that the sensitizing effect would be modified under different conditions, yet the observed robust changes, particularly regarding the blunted insulin sensitivity of the skeletal muscle tissue, would most likely not be affected. Further studies should address in detail the mechanisms and pathways, through which the *Zbtb16* mediates the sensitization to DEX-induced IR of skeletal muscle. Also, the relevance of our findings for human condition needs to be validated. So far, there is not any pharmacogenetic study involving variation in human ZBTB16 gene available. In a study of 1517 non-diabetic subjects without hypolipidemic treatment, several single nucleotide polymorphisms in *ZBTB16* gene were associated with adiposity measures, LDL, and total cholesterol (45), suggesting the importance of ZBTB16 for metabolic syndrome in humans. The metabolic disturbances including impaired glucose tolerance, dyslipidemia, and IR of skeletal muscle observed after DEX treatment in the congenic SHR-*Lx*.PD5^PD-^*^Zbtb16^* reveal the *Zbtb1*6 locus as a possible sensitizing factor for side effects of GC therapy.

## Ethics Statement

All experiments were performed in agreement with the Animal Protection Law of the Czech Republic (311/1997) which is in compliance with the European Community Council recommendations for the use of laboratory animals 86/609/ECC and were approved by the Ethical committee of the First Faculty of Medicine and the Ministry of Education, Youth and Sports of the Czech Republic (protocol ID MSMT-1461/2015-17).

## Author Contributions

VK and DK derived the new congenic substrains. OS, LS, MK, AK, and LK carried out the experimental components of the study and drafted the manuscript. FL participated in the design of the study and performed the statistical analysis. OS, VK, and LK conceived the study, participated in its design and coordination, and helped to draft the manuscript. All the authors participated in the manuscript preparation and read and approved the final manuscript.

## Conflict of Interest Statement

The authors declare that the research was conducted in the absence of any commercial or financial relationships that could be construed as a potential conflict of interest.

## Supplementary Material

Figure S1 Representative genotyping results. The results of electrophoretic separation of polymerase chain reaction products of polymorphic markers are shown for individual rat strains (primer sequences and genomic positions of individual markers are shown in Table S1 in Supplementary Material) as follows: lane 1: ladder; lanes 2–4: PD/Cub; lanes 5–7: SHR-*Lx*.PD5; lanes 8–10: SHR-*Lx*.PD5^PD-^*^Zbtb16^*; lanes 11–13: SHR-*Lx*.PD5^SHR-^*^Zbtb16^*; lanes 14–16: SHR; and lane 17: negative control.
