# Supplementary figures and images for: Single-Gene Congenic Strain Reveals the Effect of Zbtb16 on Dexamethasone-Induced Insulin Resistance
Source: Front Endocrinol (Lausanne). 2018 Apr 20;9:185. doi: 10.3389/fendo.2018.00185 (PMC5919955; doi:10.3389/fendo.2018.00185)

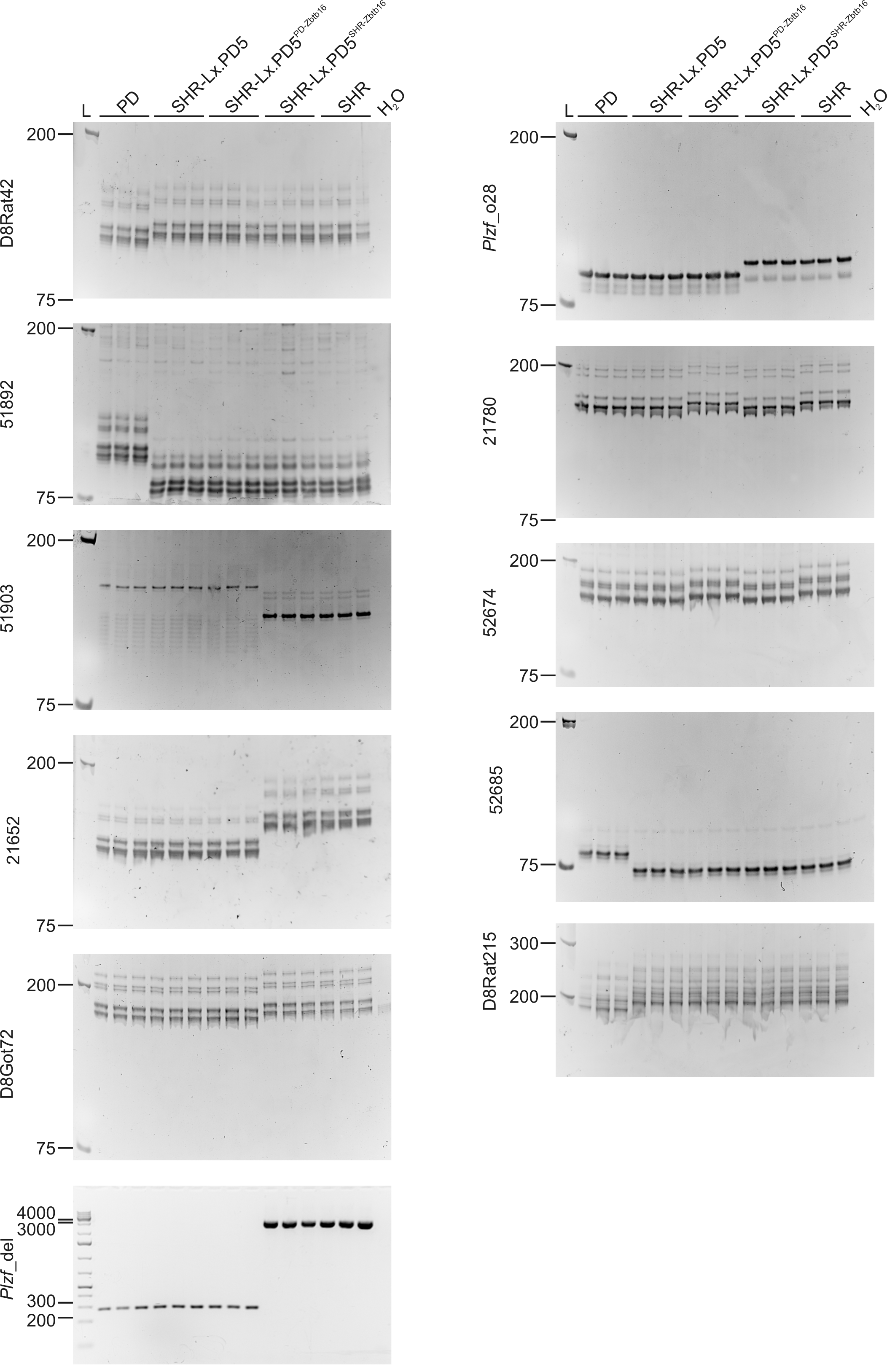

Supplement: Figure S1 — Representative genotyping results. The results of electrophoretic separation of polymerase chain reaction products of polymorphic markers are shown for individual rat strains (primer sequences and genomic positions of individual markers are shown in Table S1 in Supplementary Material) as follows: lane 1: ladder; lanes 2–4: PD/Cub; lanes 5–7: SHR-Lx.PD5; lanes 8–10: SHR-Lx.PD5PD-Zbtb16; lanes 11–13: SHR-Lx.PD5SHR-Zbtb16; lanes 14–16: SHR; and lane 17: negative control. [file image_1.TIF]
